# Supplementary material for: Oxygen-Sensitive Photo- and Radioluminescent Polyurethane Nanoparticles Modified with Octahedral Iodide Tungsten Clusters
Source: Nanomaterials (Basel). 2022 Oct 12;12(20):3580. doi: 10.3390/nano12203580 (PMC9608458; doi:10.3390/nano12203580)
Supplement: Supplementary file 1 [file nanomaterials-12-03580-s001.zip › nanomaterials-1938299-supplementary.pdf]

nanomaterials

Supplementary Materials

Article

Oxygen-Sensitive Photo- and Radioluminescent Polyurethane Nanoparticles Modified with Octahedral Iodide Tungsten Clusters

Vyacheslav A. Bardin [1], Yuri A. Vorotnikov [1,*], Dmitri V. Stass [2,3], Natalya A. Vorotnikova [1,*] and Michael A. Shestopalov [1]

[1] Nikolaev Institute of Inorganic Chemistry SB RAS, 3 Acad. Lavrentiev Ave., 630090 Novosibirsk, Russia
[2] Voevodsky Institute of Chemical Kinetics and Combustion SB RAS, 3 Institutskaya St., 630090 Novosibirsk, Russia
[3] Department of Physics, Novosibirsk State University, 2 Pirogova St., 630090 Novosibirsk, Russia
* Correspondence: vorotnikov@niic.nsc.ru (Y.A.V.); vorotnikova@niic.nsc.ru (N.A.V.)

Citation: Bardin, V.A.; Vorotnikov, Y.A.; Stass, D.V.; Vorotnikova, N.A.; Shestopalov, M.A. Oxygen-Sensitive Photo- and Radioluminescent Polyurethane Nanoparticles Modified with Octahedral Iodide Tungsten Clusters. *Nanomaterials* **2022**, *12*, 3580. https://doi.org/10.3390/nano12203580

Academic Editor: Hideya Kawasaki

Received: 12 September 2022
Accepted: 10 October 2022
Published: 12 October 2022

Publisher's Note: MDPI stays neutral with regard to jurisdictional claims in published maps and institutional affiliations.

Copyright: © 2022 by the authors. Licensee MDPI, Basel, Switzerland. This article is an open access article distributed under the terms and conditions of the Creative Commons Attribution (CC BY) license (https://creativecommons.org/licenses/by/4.0/).

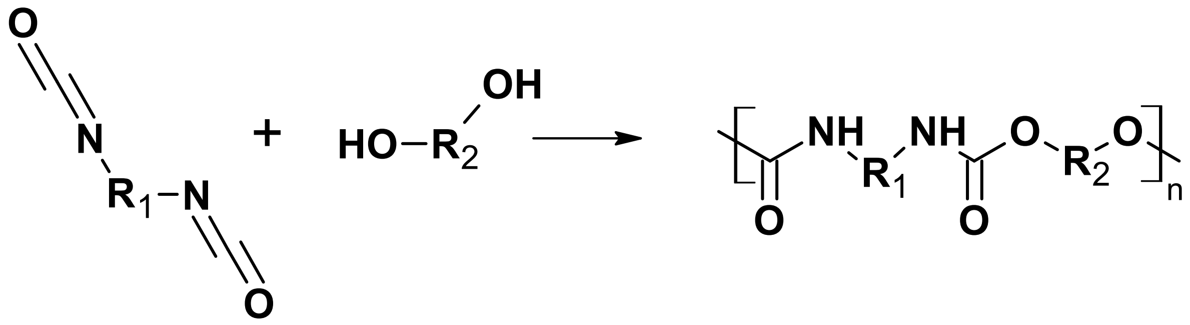

Figure S1. Scheme of polyurethane polymerisation.

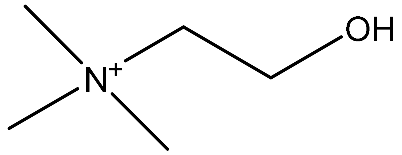

Figure S2. Structure representation of choline.

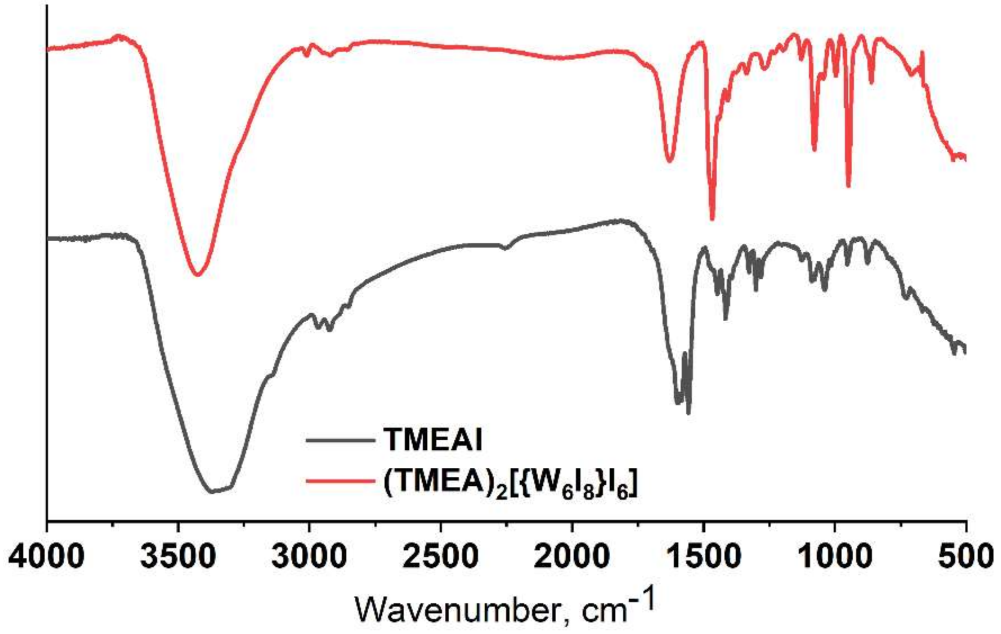

Figure S3. IR spectra for TMEAI and cluster **1.**

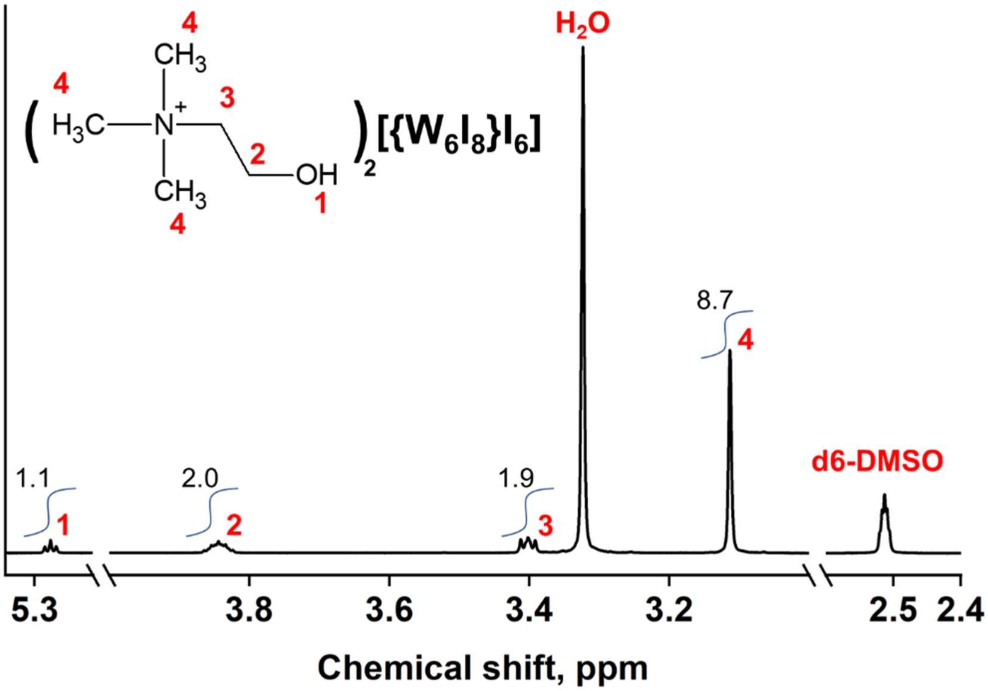

Figure S4. ^{1}H-NMR spectra for $(TMEA)_2[\{W_6I_8\}I_6]$.

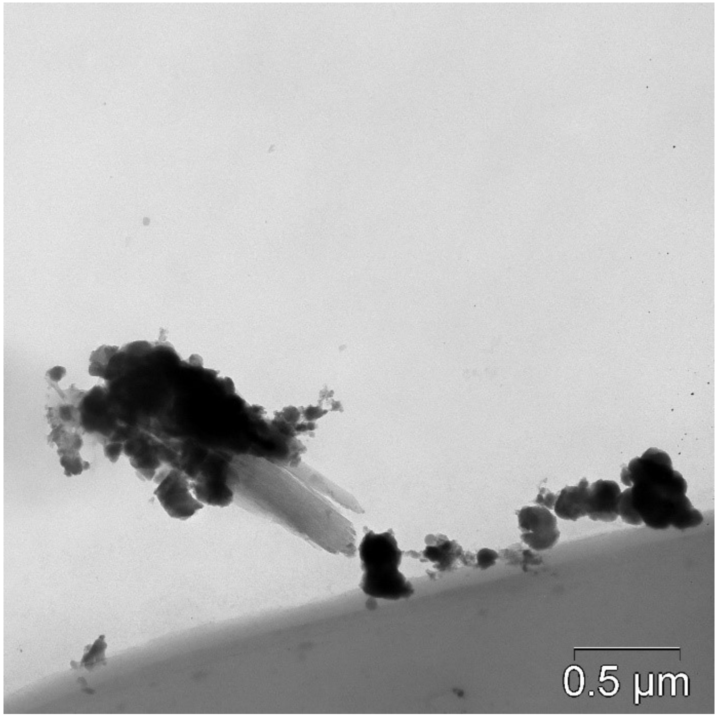

Figure S5. TEM picture of **1**^{1}@pU.

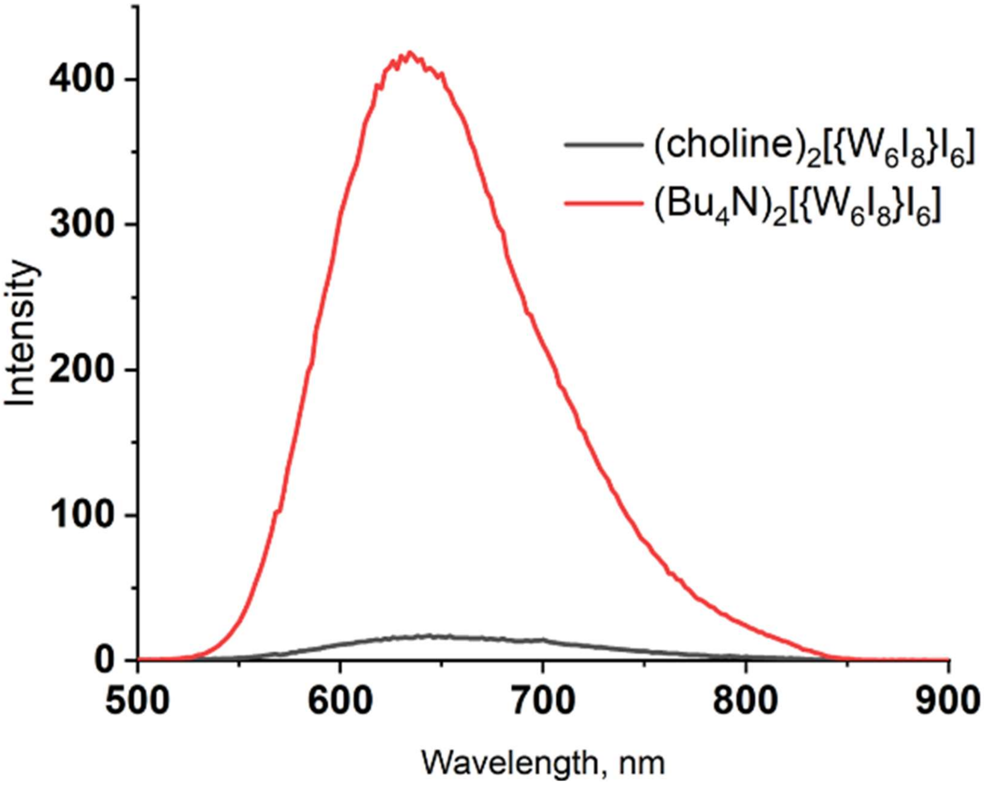

Figure S6. Comparison of luminescence spectra of tungsten clusters $(choline)_2[\{W_6I_8\}I_6]$ and $(Bu_4N)_2[\{W_6I_8\}I_6]$ in solid state, λ_{ex} = 350 nm, 5 × 5.

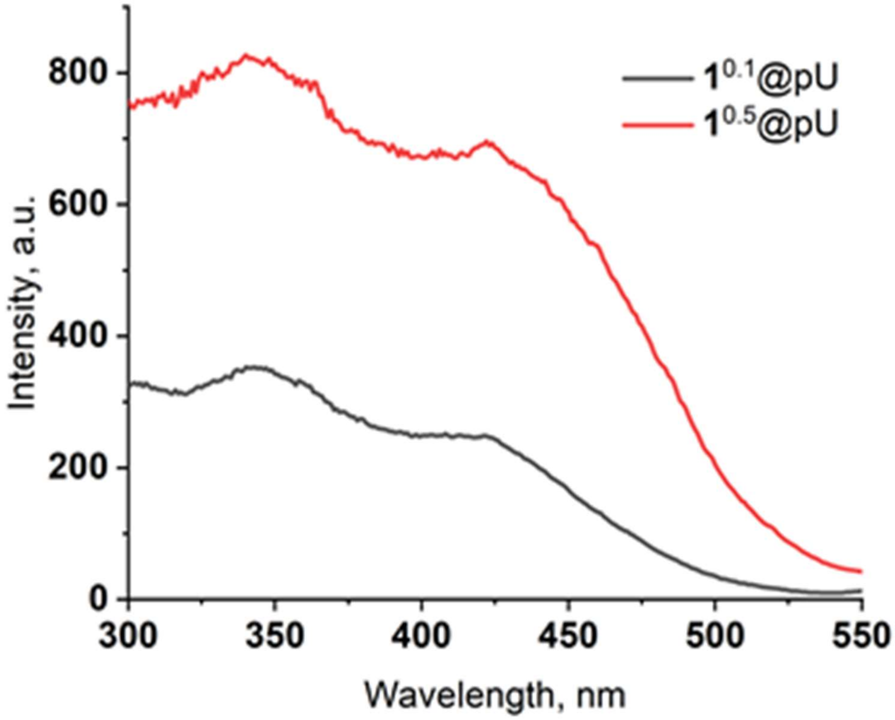

Figure S7. Excitation spectra for $\mathbf{1}^{0.1}$@pU and $\mathbf{1}^{0.5}$@pU in solid state, λ_{em} =6 45 nm.

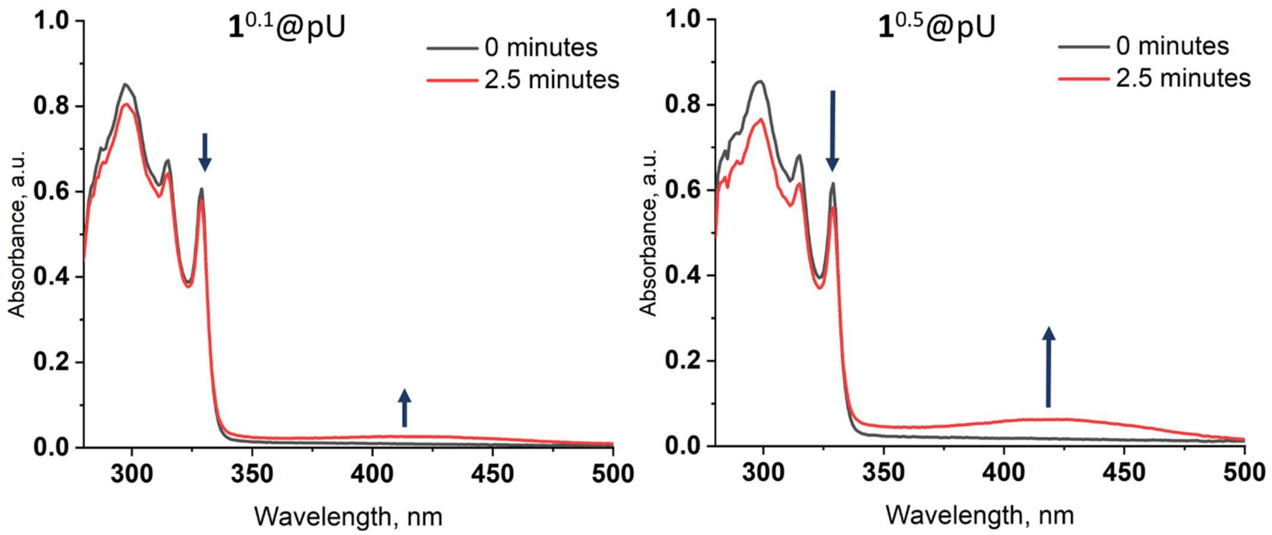

Figure S8. Characteristic UV-vis spectra of aqueous solutions (20 mL) of DHN (0.1 mM) in the presence of particles (0.05 mg mL^{-1}) **1**$^{0.1}$@pU and **1**$^{0.5}$@pU before and after white light irradiation.

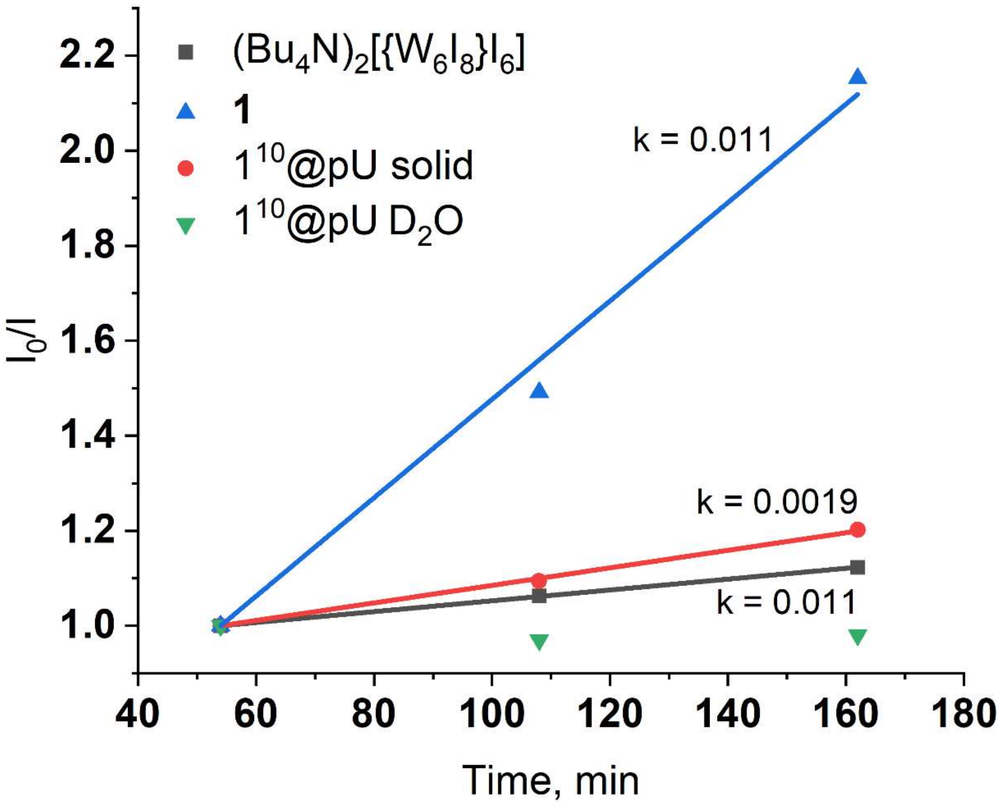

Figure S9. I_0/I vs irradiation time for XEOL spectra of molybdenum glass tube with D_2O and neat pU dispersion in D_2O.

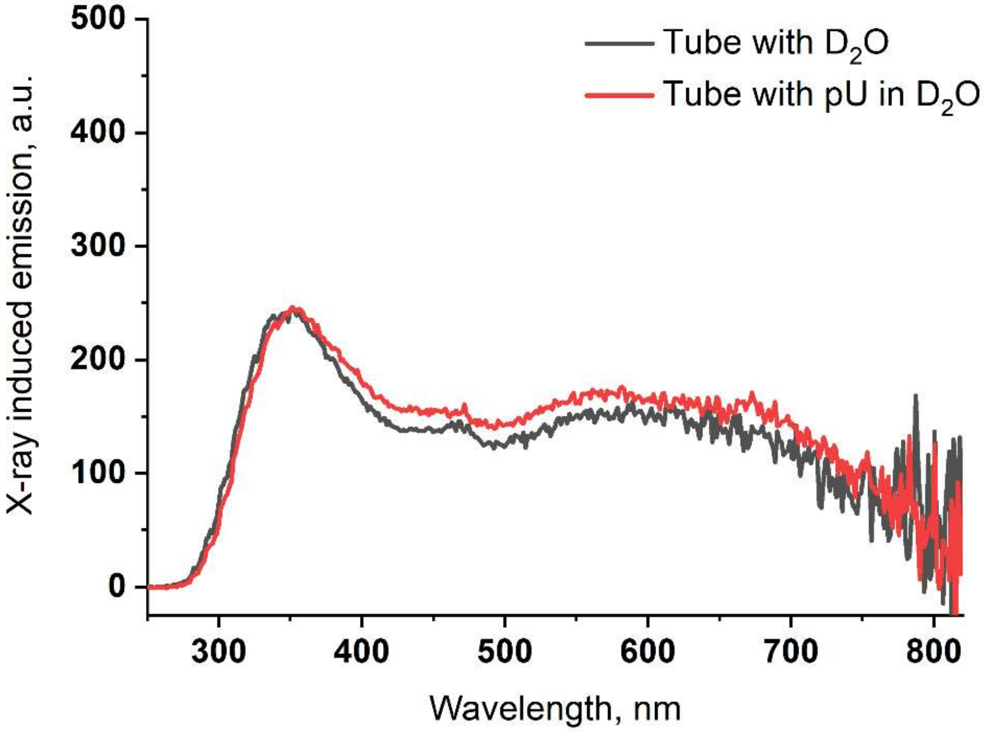

Figure S10. XEOL spectra of molybdenum glass tube with D_2O and neat pU dispersion in D_2O.
